# Prognostic value of the inflammatory prognostic index for postoperative recurrence in non-muscle-invasive bladder cancer

**DOI:** 10.1186/s12894-026-02301-3

**Published:** 2026-08-07

**Authors:** GengXu Li, Chao Tan, JunLi Wei, ShuBo Chen

**Affiliations:** Department of Urology, Xingtai People’s Hospital, Xingtai, Hebei China

**Keywords:** Non-muscle-invasive bladder cancer, Inflammatory prognostic index, Transurethral resection of bladder tumour, Recurrence-free survival, Prognosis

## Abstract

**Background:**

To evaluate the prognostic value of the inflammatory prognostic index (IPI) for recurrence after transurethral resection of bladder tumour (TURBT) in non-muscle-invasive bladder cancer (NMIBC), and to compare its discriminatory performance with that of the neutrophil-to-lymphocyte ratio (NLR), platelet-to-lymphocyte ratio (PLR), and lymphocyte-to-monocyte ratio (LMR).

**Methods:**

This retrospective study included 123 patients who underwent TURBT for primary NMIBC. The primary analysis evaluated IPI as a continuous variable. Optimal cut-off values were determined by receiver operating characteristic (ROC) curve analysis, recurrence-free survival (RFS) was characterised by Kaplan–Meier estimation, and independent predictors were identified by Cox proportional hazards regression. Bootstrap internal validation (B = 1000) was applied to quantify overfitting and assess cut-off stability.

**Results:**

During a median follow-up of 36 months, 44 patients (35.8%) developed disease recurrence. Among the markers tested, IPI showed the largest area under the curve (AUC = 0.790, 95% CI 0.705–0.874) and performed better than NLR (AUC = 0.613), PLR (AUC = 0.523, 95% CI 0.418–0.629), and LMR (AUC = 0.624). When analysed as a continuous variable, each 0.1‑unit increase in IPI was associated with a 12% higher recurrence risk (HR 1.12, 95% CI 1.07–1.18, *P* < 0.001). For clinical interpretability, the optimal IPI cut-off was 0.223. In multivariable analysis, IPI ≥ 0.223 (HR 5.574, 95% CI 2.583–12.029, *P* < 0.001) and diabetes mellitus (HR 2.853, 95% CI 1.279–6.366, *P* = 0.010) were independently associated with recurrence. IPI remained significant after adjustment for either pathological grade or EAU risk group. A pre‑specified Fine–Gray competing‑risks sensitivity simulation gave similar results, suggesting that the association was not driven by overestimation of recurrence in the presence of hypothetical competing non‑cancer mortality (subdistribution HR 5.91, 95% CI 2.87–12.19; *P* < 0.001). Bootstrap validation suggested limited overfitting (bias-corrected C-statistic 0.790; optimism 0.020), a stable cut-off (bootstrap median 0.227, 95% CI 0.084–0.617), and good calibration (bias-corrected calibration slope 0.86, Brier score 0.091).

**Conclusions:**

Preoperative IPI, analysed primarily as a continuous variable, was an independent predictor of recurrence after TURBT in patients with NMIBC, with each 0.1‑unit increase associated with a 12% higher recurrence risk. It showed better discriminatory accuracy than NLR, PLR, and LMR and may be a practical, low‑cost adjunct for risk stratification and individualized postoperative surveillance; the dichotomised threshold reported here is cohort‑specific and requires external validation.

**Supplementary Information:**

The online version contains supplementary material available at https://doi.org/10.1186/s12894-026-02301-3.

## Introduction

Bladder cancer is one of the most common urological malignancies worldwide, and about 75% of newly diagnosed cases are non-muscle-invasive bladder cancer (NMIBC) [1]. Even with TURBT followed by intravesical instillation therapy, recurrence remains frequent, with five-year recurrence rates reported at 44–58% [2, 3]. The EAU 2021 risk stratification model uses established clinicopathological factors, but predicting recurrence for an individual patient is still challenging. Additional objective biomarkers may therefore help refine postoperative risk assessment [4].

Systemic inflammation is closely linked to tumour behaviour. A growing body of evidence suggests that pro-inflammatory microenvironments can promote tumour progression and weaken host immune responses [5, 6]. Several blood-based indices, including NLR, PLR, and LMR, have been studied in this context. However, their value for predicting NMIBC outcomes remains uncertain, as studies in different patient cohorts have produced inconsistent results [7–11]. The inflammatory prognostic index (IPI), calculated as C-reactive protein × (neutrophil/lymphocyte) / albumin, combines information on the acute-phase response, immune balance, and nutritional reserve. Elevated IPI has been associated with poor prognosis in several solid tumours, including lung, gastric, colorectal, and renal cancers [12–15]. More recently, Mokbel et al. developed and externally validated a urothelial-specific variant (U-IPI), showing that this composite index can predict responses to immune checkpoint blockade in locally advanced or metastatic urothelial carcinoma [16]. These findings support the relevance of IPI-related measures in urothelial cancer, but their role in earlier-stage NMIBC has not been well defined.

In this study, we examined the association between preoperative IPI and recurrence after TURBT in patients with NMIBC. We also compared the predictive performance of IPI with that of the commonly used single-ratio markers NLR, PLR, and LMR.

## Methods

### Study population

We retrospectively analysed consecutive patients who underwent initial TURBT at Xingtai People’s Hospital between August 2020 and January 2023 and were pathologically diagnosed with primary NMIBC (Ta, T1, or Tis). Eligibility criteria included: (1) complete haematological and biochemical data obtained within one week prior to surgery; (2) completion of at least the induction phase of intravesical therapy as prescribed per the EAU risk-adapted protocol—specifically, a single immediate instillation for low-risk patients, an induction course of at least four weekly instillations for intermediate-risk patients, and completion of the initial six-week BCG induction course for high-risk patients, as verified by treatment records; and (3) availability of complete follow-up data. Exclusion criteria were: concurrent malignancy at any other primary site; active infection or use of antibiotics, corticosteroids, or immunosuppressants within two weeks before surgery; and pre-existing haematological disorders, autoimmune diseases, or severe hepatic or renal insufficiency. Postoperative intravesical instillation followed EAU guideline-based risk stratification: low-risk patients received single-dose chemotherapy, intermediate-risk patients received induction chemotherapy with or without maintenance, and high-risk patients received BCG immunotherapy for at least one year unless it was contraindicated or declined.

### Data collection and definitions

Baseline demographic information, smoking history, comorbidities (hypertension and diabetes mellitus), and preoperative laboratory data were collected from electronic medical records. Laboratory variables included absolute neutrophil, lymphocyte, monocyte, and platelet counts, C-reactive protein (CRP; mg/dL), and serum albumin (g/dL). The indices were calculated as follows: NLR = neutrophil/lymphocyte; PLR = platelet/lymphocyte; LMR = lymphocyte/monocyte; and IPI = CRP × NLR / albumin. Tumour-related variables included tumour number, maximum diameter, T stage, and pathological grade according to the WHO 2004 classification. Risk groups were assigned according to the 2021 EAU guidelines.

### Follow-up

Follow-up cystoscopy was performed every three months during the first two years and every six months thereafter, together with annual upper urinary tract imaging. The median follow-up was 36 months (range: 3–66 months). RFS was defined as the time from surgery to the first pathologically confirmed recurrence or the last follow-up visit (31 January 2026), whichever came first.

### Statistical analysis

#### Conventional statistical methods

The primary analysis evaluated IPI as a continuous variable; the Youden-index-derived cut-off was used for secondary, clinically interpretable analyses. Statistical analyses were performed with SPSS version 27.0 (IBM Corp., Armonk, NY, USA). Continuous variables were tested for normality using the Shapiro–Wilk test. Normally distributed variables are presented as mean ± SD and were compared with the independent-samples t-test. Non-normally distributed variables are shown as median (IQR) and were compared with the Mann–Whitney U test. Categorical variables are reported as frequencies and percentages and were analysed using the Pearson chi-square test or Fisher’s exact test, as appropriate.

#### Cut-off determination and survival analysis

For each inflammatory index, the optimal dichotomisation threshold was selected from ROC curve analysis using the Youden index (sensitivity + specificity − 1). Time-to-event outcomes were estimated with the Kaplan–Meier method, and RFS differences between groups were tested using the log-rank test.

#### Multivariable Cox regression

Cox proportional hazards models were used for multivariable prognostic analysis. Variables with *P* < 0.10 in univariate screening were considered for inclusion. Because IPI includes the neutrophil-to-lymphocyte component and therefore overlaps mathematically with NLR, the two indices were not entered into the same model. Instead, three parallel multivariable models were built, each combining the clinicopathological covariates with one inflammatory index (IPI, NLR, or LMR). PLR was not included in the multivariable analysis because it showed no significant discriminatory ability on ROC analysis (AUC = 0.523, *P* = 0.669) and was not associated with recurrence in univariate Cox regression (HR = 0.999, 95% CI 0.995–1.003, *P* = 0.656). To further test whether IPI was independent of established pathological stratification, two pre-specified sensitivity models included IPI together with either histological grade (Model A) or EAU risk category (Model B). The primary multivariable model included 44 recurrence events and six covariates, giving an events-per-variable ratio of about 7.3, within the acceptable range of 5–9 events per variable for Cox regression [17].

#### Bootstrap internal validation

Bootstrap internal validation (B = 1000) was used to examine possible overfitting related to the Youden-index-derived IPI cut-off. The analysis followed the optimism-correction framework described by Harrell et al. [18]. Harrell’s C-statistic was calculated in each bootstrap sample and in the original dataset. Mean optimism across resamples was then subtracted from the apparent C-statistic to obtain the bias-corrected estimate. Bootstrap percentile 95% confidence intervals for the IPI hazard ratio were taken from the empirical distribution of the 1000 estimates. Cut-off stability was evaluated by recording the Youden-optimal IPI threshold in each bootstrap resample and reporting the median and 95% percentile confidence interval.

#### Pre‑specified competing‑risks sensitivity simulation

Because this cohort was relatively elderly (mean age 69.3 years), standard Kaplan–Meier estimates may overstate cumulative recurrence if non‑cancer death competes with the event of interest. As all‑cause mortality data were not systematically collected in this retrospective study, a pre‑specified sensitivity simulation was performed using the Fine–Gray subdistribution hazard model [19], with non‑cancer death treated as the competing event. Competing‑event status was imputed from age‑specific all‑cause mortality rates for China derived from the Global Burden of Disease Study 2021 (GBD 2021) demographic estimates [20]. This analysis is explicitly hypothetical and is intended to explore whether competing mortality could materially bias the conventional Kaplan–Meier estimates; it does not substitute for a formal competing risks analysis with observed events. The imputation was repeated across 200 Monte Carlo resamples, and Fine–Gray estimates were pooled using Rubin’s combining rules.

#### Model calibration

Calibration was assessed at 36 months using bootstrap-based calibration curves, calibration slope, and Brier score. The calibration slope was estimated from 1000 bootstrap resamples. The Brier score was calculated with the riskRegression package, which was also used to generate the calibration curve through 1000-fold bootstrap cross-validation. Given the modest sample size, the analytical framework prioritised a single pre-specified primary analysis (continuous IPI), with all other analyses (dichotomisation, RCS, bootstrap, calibration, and the competing-risks simulation) reported as supportive or exploratory rather than as independent confirmatory evidence.

## Results

### Patient characteristics

A total of 123 patients were included (98 males, 79.7%; mean age 69.3 ± 11.8 years). Over a median follow-up of 36 months, 44 patients (35.8%) experienced recurrence. Patients in the recurrence group were older (72.32 ± 10.36 vs. 67.67 ± 12.18 years, *P* = 0.035) and more often had diabetes mellitus (22.7% vs. 8.9%, *P* = 0.033). Sex, smoking history, and hypertension were similar between groups (Table 1). Recurrent tumours were more often multiple, T1 stage, high grade, and high-risk (all *P* < 0.05). Inflammatory markers also differed: NLR and IPI were higher in the recurrence group, LMR was lower, and PLR showed no significant between-group difference (Table 2). Of the 123 patients, 7 (5.7%) had carcinoma in situ (Tis), either pure Tis (*n* = 3) or Tis with concurrent Ta/T1 (*n* = 4). All 7 patients with Tis were classified as high‑risk and received BCG induction. The median IPI in the Tis subgroup was 0.72 (IQR 0.24–1.85), and 5 of the 7 had an IPI ≥ 0.223.

**Table 1:** Comparison of general demographic and clinical characteristics between patients with and without recurrence

Parameter	Non-recurrence (*n* = 79)	Recurrence (*n* = 44)	*P* value
Age (years, mean ± SD)	67.67 ± 12.18	72.32 ± 10.36	**0.035** ^*^
Sex, male, n (%)	61 (77.2%)	37 (84.1%)	0.364
Smoking history, n (%)	35 (44.3%)	23 (52.3%)	0.396
Hypertension, n (%)	39 (49.4%)	27 (61.4%)	0.201
Diabetes mellitus, n (%)	7 (8.9%)	10 (22.7%)	**0.033** ^*^

Data are presented as mean ± SD or n (%). *P* values were calculated using the independent-samples t-test for age and the chi-square test for categorical variables. Age distributions did not significantly deviate from normality in either group (Shapiro–Wilk: W = 0.982, P = 0.322 for non-recurrence; W = 0.968, P = 0.247 for recurrence), supporting the use of parametric testing

*P < 0.05 was considered statistically significant

**Table 2:** Comparison of clinicopathological characteristics and inflammatory markers between patients with and without recurrence

Parameter	Non-recurrence (*n* = 79)	Recurrence (*n* = 44)	*P* value
Inflammatory markers			
NLR, median (IQR)	2.12 (1.41, 2.67)	2.45 (1.87, 4.08)	**0.038** ^*^
PLR, median (IQR)	129.03 (102.75, 163.38)	130.19 (103.78, 164.97)	0.669
LMR, median (IQR)	4.75 (3.91, 6.67)	4.25 (2.85, 5.37)	**0.022** ^*^
IPI, median (IQR)	0.060 (0.027, 0.177)	0.670 (0.189, 1.757)	**< 0.001** ^*^
CRP, mg/dL, median (IQR)	0.11 (0.06, 0.32)	1.08 (0.35, 1.99)	**< 0.001** ^*^
Albumin, g/dL, median (IQR)	3.94 (3.75, 4.21)	3.95 (3.51, 4.13)	0.416
Tumour characteristics			
Tumour size, cm, median (IQR)	2.20 (1.60, 2.80)	2.50 (1.53, 3.85)	0.213
Multiple tumours, n (%)	27 (34.2%)	24 (54.5%)	**0.028** ^*^
T1 stage, n (%)	36 (45.6%)	32 (72.7%)	**0.004** ^*^
Tis present, n (%)	3 (3.8%)	4 (9.1%)	0.419
High-grade, n (%)	25 (31.6%)	29 (65.9%)	**< 0.001** ^*^
High-risk group, n (%)	39 (49.4%)	33 (75.0%)	**0.006** ^*^

Continuous variables are presented as median (IQR) and were compared using the Mann–Whitney U test. Categorical variables are shown as n (%) and were compared with the chi-square test or Fisher’s exact test

*CRP* C-reactive protein, *NLR* Neutrophil-to-lymphocyte ratio, *PLR* Platelet-to-lymphocyte ratio, *LMR* Lymphocyte-to-monocyte ratio, *IPI* Inflammatory prognostic index

*P < 0.05 was considered statistically significant. T stage categories are not mutually exclusive: 4 patients had concurrent Tis with Ta or T1 disease and are counted in both the relevant Ta/T1 category and the “Tis present” row; 3 patients had pure Tis

### Cut-off determination and predictive accuracy

**Fig. 1:**
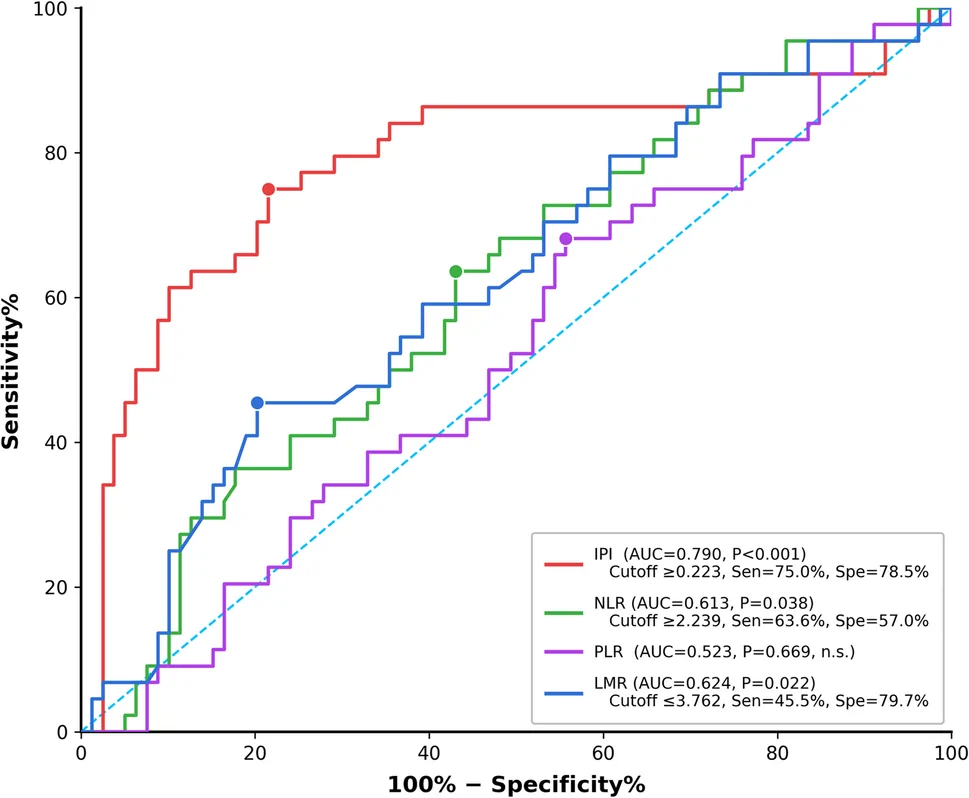
ROC curves for IPI, NLR, PLR, and LMR in predicting postoperative recurrence. IPI showed the highest AUC (0.790, 95% CI 0.705–0.874). PLR was not statistically significant (AUC = 0.523, *P* = 0.669). The optimal cut-offs were IPI ≥ 0.223, NLR ≥ 2.239, and LMR ≤ 3.762

The ROC-derived AUC for IPI was 0.790 (95% CI 0.705–0.874), which was higher than the AUCs for NLR (0.613), LMR (0.624), and PLR (0.523; *P* = 0.669). DeLong’s test showed that IPI performed significantly better than NLR (AUC difference = 0.177, z = 3.458, *P* = 0.001), PLR (AUC difference = 0.267, z = 4.399, *P* < 0.001), and LMR (AUC difference = 0.415, z = 4.855, *P* < 0.001). Based on the Youden index, the optimal cut-offs were IPI ≥ 0.223 (sensitivity 75.0%, specificity 78.5%), NLR ≥ 2.239 (sensitivity 63.6%, specificity 57.0%), and LMR ≤ 3.762 (sensitivity 79.7%, specificity 45.5%) (Fig. 1). The primary statistical assessment of IPI was based on the continuous variable; the Youden-derived cut-off of 0.223 was used only for secondary, clinically oriented analyses.

### Association with clinicopathological features

High IPI (≥ 0.223) was associated with older age, larger tumours, multiple tumours, T1 stage, high grade, and high-risk classification (all *P* < 0.05). By contrast, high NLR was associated only with diabetes mellitus, larger tumour size, and T1 stage (Table 3). LMR was associated with T1 stage and high grade, but it did not independently predict recurrence (Table 3).

**Table 3:** Association of IPI and NLR with clinicopathological characteristics

Parameter	Low IPI (*n* = 73)	High IPI (*n* = 50)	*P* (IPI)	Low NLR (*n* = 61)	High NLR (*n* = 62)	*P* (NLR)
Age (years, mean ± SD)	67.25 ± 12.5	72.38 ± 9.72	**0.017** ^*^	68.56 ± 12.23	70.10 ± 11.28	0.469
BMI (kg/m², mean ± SD)	24.91 ± 3.57	24.64 ± 3.83	0.688	25.02 ± 4.26	24.58 ± 2.99	0.510
Diabetes mellitus, n (%)	8 (11.0%)	9 (18.0%)	0.266	4 (6.6%)	13 (21.0%)	**0.021** ^*^
Tumour size (cm), median (IQR)	2.20 (1.40, 2.70)	2.60 (1.70, 3.50)	**0.014** ^*^	2.00 (1.20, 3.00)	2.50 (2.00, 3.30)	**0.018** ^*^
Multiple tumours, n (%)	24 (32.9%)	27 (54.0%)	**0.020** ^*^	22 (36.1%)	29 (46.8%)	0.228
T1 stage, n (%)	32 (43.8%)	36 (72.0%)	**0.002** ^*^	28 (45.9%)	40 (64.5%)	**0.038** ^*^
High-grade, n (%)	23 (31.5%)	31 (62.0%)	**0.003** ^*^	24 (39.3%)	30 (48.4%)	0.575
High-risk group, n (%)	35 (47.9%)	37 (74.0%)	**0.008** ^*^	32 (52.5%)	40 (64.5%)	0.354

Cut-offs were IPI ≥ 0.223 and NLR ≥ 2.239. Data are presented as mean ± SD, median (IQR), or n (%)

*IPI* Inflammatory prognostic index, *NLR* Neutrophil-to-lymphocyte ratio, *BMI* Body mass index, *IQR* Interquartile range

*P < 0.05 was considered statistically significant

### Survival analysis

**Fig. 2:**
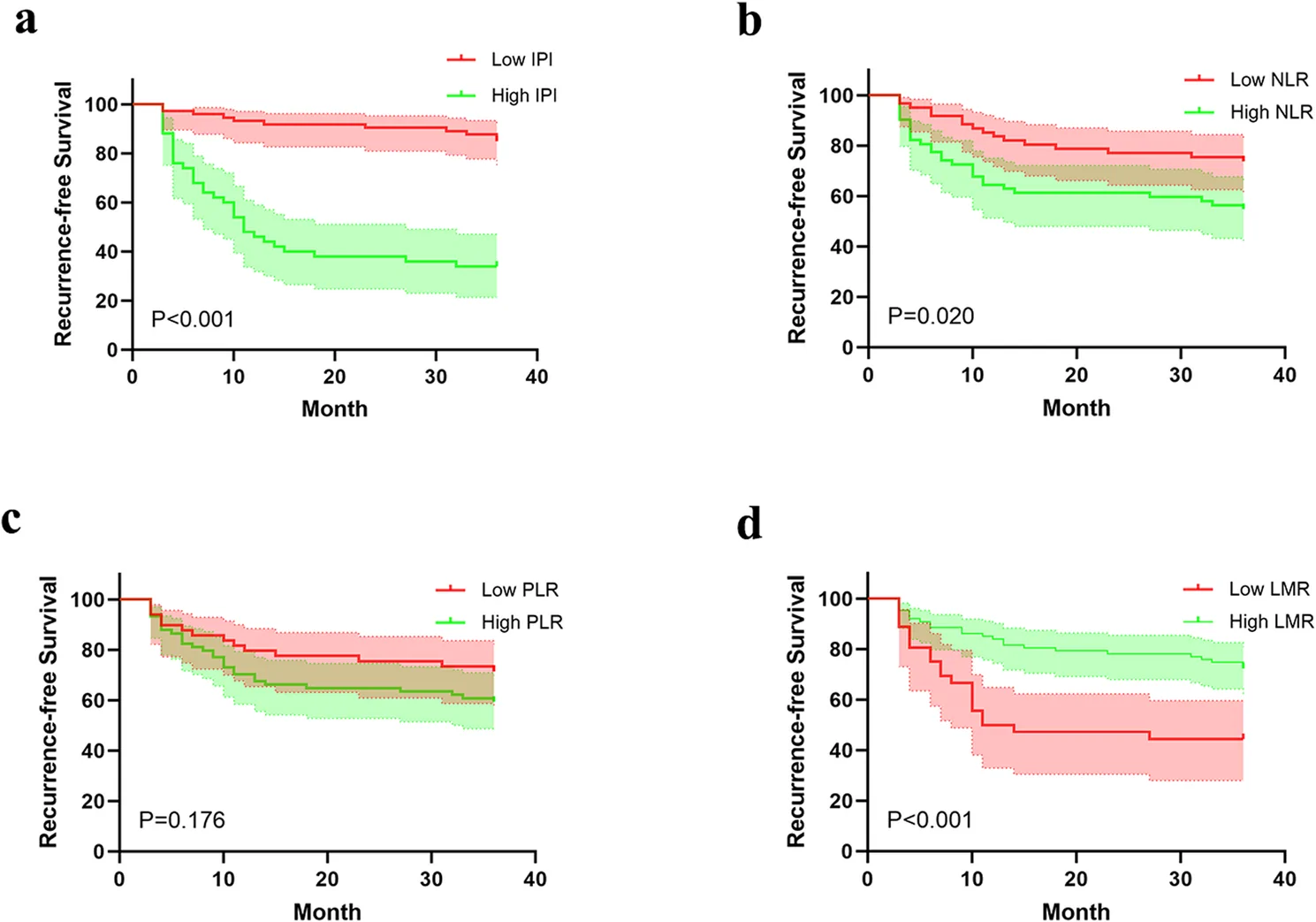
Kaplan–Meier curves for recurrence-free survival stratified by (**a**) IPI, (**b**) NLR, (**c**) PLR, and (**d**) LMR. High IPI, high NLR, and low LMR were associated with worse RFS (all *P* < 0.05), whereas PLR stratification showed no significant difference (*P* = 0.176)

Kaplan–Meier analysis showed shorter RFS in the high-IPI group than in the low-IPI group (mean 18.04 ± 1.98 vs. 33.36 ± 0.96 months, log-rank *P* < 0.001). RFS was also shorter in the high-NLR group (*P* = 0.020) and the low-LMR group (*P* < 0.001). PLR-based stratification did not show a significant RFS difference (*P* = 0.176). Survival curves are shown in Fig. 2.

### IPI as a continuous variable and dose–response analysis

**Fig. 3:**
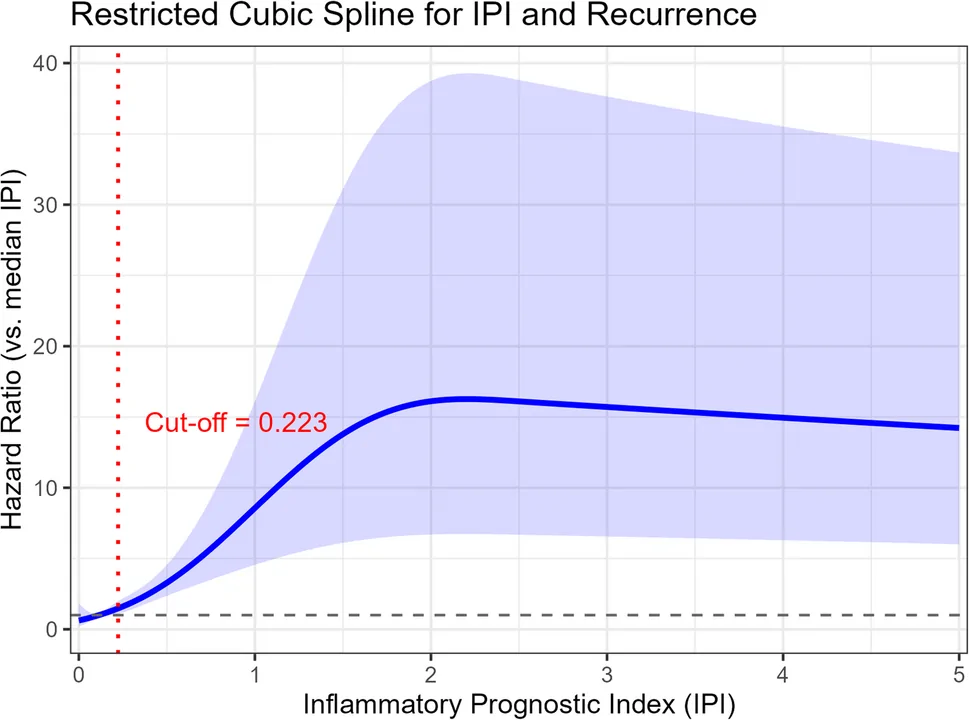
Restricted cubic spline plot of the dose–response relationship between IPI and recurrence. The solid blue line represents the hazard ratio (HR) relative to the median IPI (reference), with the shaded band indicating the 95% confidence interval. The dashed horizontal line marks HR = 1. The red dotted vertical line indicates the optimal cut‑off value (0.223) derived from ROC analysis. For visualization, the X‑axis is truncated at IPI = 5, covering the range where the vast majority of observations lie. A single extreme outlier (IPI = 97.8) was omitted from the plot for visual clarity but was included in all statistical analyses; a sensitivity analysis excluding this observation yielded nearly identical results (Supplementary Table S1). Knots were placed at the 5th, 35th, 65th, and 95th percentiles of the original IPI distribution. *P* for non‑linearity < 0.0001

In the primary analysis with IPI modelled as a linear continuous variable, each 0.1‑unit increase was associated with a 12% higher recurrence risk (HR = 1.12, 95% CI 1.07–1.18, *P* < 0.001). Because the relationship was found to be non‑linear, we also applied restricted cubic spline (RCS) analysis. As shown in Fig. 3, recurrence risk increased slowly at low IPI values but rose steeply once IPI exceeded approximately 0.2, consistent with a threshold effect (P for non‑linearity < 0.0001). The 95% confidence band widened at higher IPI levels due to sparse observations. For visual clarity, the X‑axis in Fig. 3 is truncated at IPI = 5 (the 99th percentile); a single extreme outlier (IPI = 97.8) is not displayed. In a pre‑specified sensitivity analysis that excluded this outlier, the continuous HR for IPI remained essentially unchanged (HR per 0.1‑unit increase: 1.13, 95% CI 1.07–1.19, *P* < 0.001), and the ROC‑derived optimal cut‑off was unchanged (0.223 in both analyses). Thus, the extreme value did not materially influence the estimates. Given the non‑linear pattern, the dichotomised IPI (≥ 0.223) is presented subsequently as a secondary, clinically interpretable analysis; however, the cut‑off is data‑derived and requires external validation.

### Univariate and multivariate Cox regression analysis

Univariate analysis identified age, diabetes mellitus, tumour size, tumour number, T stage, pathological grade, risk group, NLR, LMR, and IPI as predictors of recurrence. IPI had the largest univariate hazard ratio (HR 6.798). In the primary multivariable model containing IPI, both IPI ≥ 0.223 (HR = 5.574, 95% CI: 2.583–12.029, *P* < 0.001) and diabetes mellitus (HR = 2.853, 95% CI: 1.279–6.366, *P* = 0.010) remained independent risk factors. NLR and LMR were not independently significant in their respective multivariable models (*P* = 0.650 and *P* = 0.734, respectively). When treatment type (BCG vs. chemotherapy) was added to the primary Cox model, IPI ≥ 0.223 remained independently associated with recurrence (adjusted HR = 5.816, 95% CI = 2.822–11.984, *P* < 0.001), whereas instillation type was not independently associated with recurrence (HR = 0.662, 95% CI = 0.295–1.487, *P* = 0.318). These findings are summarised in Table 4.

**Table 4:** Univariate and multivariate Cox regression analysis of postoperative recurrence

Parameter	Univariate HR (95% CI), *P*	Model 1 (IPI) HR (95% CI), *P*	Model 2 (NLR) HR (95% CI), *P*	Model 3 (LMR) HR (95% CI), *P*
Age (per year)	1.029 (1.003–1.057), 0.031^*^	1.017 (0.986–1.049), 0.295	1.015 (0.981–1.049), 0.382	1.017 (0.986–1.049), 0.288
Diabetes mellitus	2.595 (1.278–5.267), 0.008^*^	2.853 (1.279–6.366), 0.010^*^	2.589 (1.273–5.267), 0.009^*^	2.374 (1.159–4.861), 0.018^*^
Tumour size (per cm)	1.309 (1.052–1.627), 0.016^*^	0.960 (0.733–1.257), 0.767	1.202 (0.967–1.494), 0.097	1.215 (0.979–1.507), 0.078
Tumour number (multiple)	1.980 (1.093–3.588), 0.024^*^	1.159 (0.617–2.180), 0.646	1.478 (0.815–2.681), 0.199	1.603 (0.885–2.902), 0.120
T stage (T1 vs. Ta)	2.770 (1.425–5.384), 0.003^*^	1.651 (0.205–13.272), 0.637	1.812 (0.842–3.897), 0.129	2.013 (0.935–4.333), 0.074
Pathological grade^a^	2.691 (1.566–4.625), < 0.001^*^	*Not included*	*Not included*	*Not included*
Risk stratification^a^	2.620 (1.413–4.857), 0.002^*^	*Not included*	*Not included*	*Not included*
NLR (high vs. low)	2.031 (1.098–3.757), 0.024^*^	—	1.193 (0.556–2.557), 0.650	—
LMR (low vs. high)^b^	0.384 (0.212–0.698), 0.002^*^	—	—	1.141 (0.533–2.444), 0.734
IPI (high vs. low)	6.798 (3.412–13.542), < 0.001^*^	5.574 (2.583–12.029), < 0.001^*^	—	—
Instillation (BCG vs. chemotherapy)	1.739 (0.909–3.326), 0.095	0.662 (0.295–1.478), 0.318	0.634 (0.288–1.393), 0.257	0.626 (0.281–1.396), 0.252

Multivariable models were fitted separately to avoid multicollinearity. Model 1 includes clinicopathological factors plus IPI; Model 2 includes clinicopathological factors plus NLR; and Model 3 includes clinicopathological factors plus LMR.

*HR* Hazard ratio, *CI* Confidence interval, *NLR* Neutrophil-to-lymphocyte ratio, *LMR* Lymphocyte-to-monocyte ratio, *IPI* Inflammatory prognostic index

*P < 0.05 was considered statistically significant

^a^Pathological grade and risk stratification were not included in the main multivariable models because of collinearity with other tumour features. ^b^The univariate HR is reported for high LMR as a protective factor, whereas the multivariable HR is reported for low LMR (cut-off < 3.762). Instillation regimen was coded as BCG = 1 and chemotherapy = 0. The HR for IPI in Model 1 is from the primary model without adjustment for instillation. After adding instillation as a covariate, the adjusted HR for IPI was 5.816 (95% CI: 2.822–11.984, *P* < 0.001)

Values in bold indicate statistical significance (*P* < 0.05)

### Sensitivity analysis

In sensitivity models that included either pathological grade (Model A) or EAU risk group (Model B), IPI ≥ 0.223 remained a strong independent predictor of recurrence (Table 5). Pathological grade and EAU high-risk classification did not reach statistical significance in these models, whereas diabetes mellitus remained independently associated with recurrence in both. Variance inflation factor (VIF) analysis showed little collinearity among IPI, pathological grade, and EAU risk group (VIF = 1.091, 2.299, and 2.251, respectively; all < 5). Thus, the lack of significance for pathological stratification variables in the combined models was unlikely to be explained by multicollinearity. These findings suggest that preoperative IPI provides prognostic information that is complementary to standard pathological stratification.

**Table 5:** Sensitivity analysis: multivariate Cox regression models testing the independent prognostic value of IPI beyond pathological stratification

Parameter	Model A (with Grade) HR (95% CI)	*P* value	Model B (with EAU Risk) HR (95% CI)	*P* value
Age (per year)	1.014 (0.985–1.045)	0.339	1.017 (0.987–1.047)	0.262
Diabetes mellitus (yes vs. no)	2.446 (1.117–5.359)	**0.025** ^*^	2.306 (1.054–5.041)	**0.036** ^*^
Tumour size (per cm)	0.979 (0.755–1.268)	0.870	1.035 (0.813–1.317)	0.782
Tumour number (multiple vs. single)	1.356 (0.730–2.521)	0.335	1.418 (0.765–2.627)	0.267
T stage (T1 vs. Ta)	1.166 (0.457–2.975)	0.748	1.746 (0.231–13.217)	0.589
Pathological grade (high vs. non-high)	1.866 (0.754–4.618)	0.177	—	—
EAU risk group (high vs. non-high)	—	—	0.994 (0.122–8.059)	0.995
IPI (≥ 0.223 vs. < 0.223)	5.176 (2.543–10.537)	**< 0.001** ^*^	5.207 (2.579–10.511)	**< 0.001** ^*^

Two sensitivity models were used to avoid collinearity. Model A includes pathological grade, and Model B includes EAU risk group

*CI* Confidence interval, *IPI* Inflammatory prognostic index, *EAU* European Association of Urology

*P < 0.05 was considered statistically significant. HR: hazard ratio

### Bootstrap internal validation

Bootstrap internal validation (B = 1000) was used to quantify overfitting and test the stability of the Youden-index-derived IPI cut-off. The apparent Harrell’s C-statistic for the primary multivariable model was 0.810. After optimism correction, the bootstrap-adjusted C-statistic was 0.790 (optimism = 0.020), indicating limited overfitting, consistent with the events-per-variable ratio of 7.3. Across the 1000 resamples, the Youden-optimal IPI threshold had a median of 0.227 (mean ± SD: 0.312 ± 0.174; 95% bootstrap CI: 0.084–0.617). Overall, 68.8% of resamples produced a threshold ≥ 0.223, supporting the stability of this dichotomisation point. The bootstrap-corrected AUC for continuous IPI was 0.792, almost identical to the apparent value (0.790), suggesting little discriminative optimism. The bootstrap percentile 95% CI for the IPI hazard ratio was 3.218–13.356; all 1000 resamples yielded HR > 1, with bootstrap *P* < 0.001. These results support the robustness of IPI as an independent predictor of recurrence.

### Model calibration

**Fig. 4:**
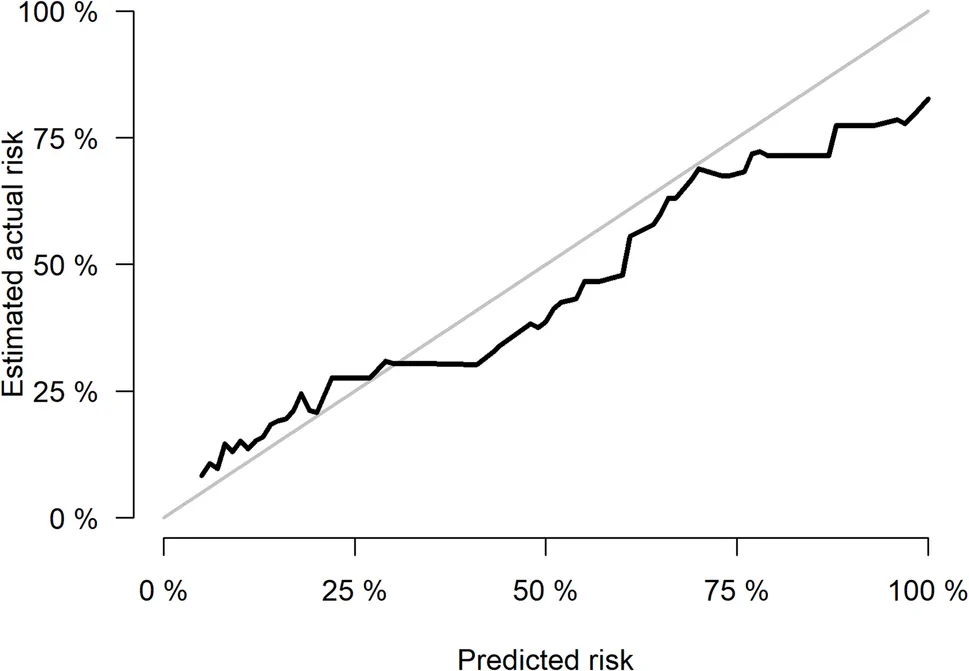
Bootstrap calibration curve at 36 months for the multivariable Cox model. The dashed diagonal line indicates perfect calibration. The bias-corrected calibration slope was 0.86

The 36-month calibration curve showed good agreement between predicted and observed recurrence-free survival probabilities (Fig. 4). The bias-corrected calibration slope from 1000 bootstrap resamples was 0.86, suggesting only modest over-optimism. The Brier score for the multivariable model was 0.091 (95% CI 0.053–0.129), significantly lower than that of the null model (ΔBrier = -0.057, *P* < 0.001), indicating good overall predictive accuracy.

### Pre‑specified competing‑risks sensitivity simulation

Given the mean age of 69.3 years in this cohort, a pre-specified competing risks sensitivity analysis was performed using the Fine–Gray subdistribution hazard model, with non-cancer death treated as the competing event. This simulation is hypothetical and intended to assess whether competing mortality could substantially bias the Kaplan–Meier estimates. Competing-event status was imputed from age-specific all-cause mortality rates for China derived from the GBD 2021 demographic estimates [20] and pooled across 200 Monte Carlo resamples.

The pooled Fine–Gray model produced a subdistribution hazard ratio (sHR) of 5.91 for IPI ≥ 0.223 (95% CI: 2.87–12.19; *P* < 0.001), which was very close to the conventional Cox HR of 5.574 (Table 4). Diabetes mellitus also remained independently associated with recurrence in the competing risks framework (sHR 2.41, 95% CI: 1.11–5.25; *P* = 0.027). Age, T stage, tumour multiplicity, and tumour size were not statistically significant in the Fine–Gray model (all *P* > 0.10), in line with the primary Cox analysis. Results are summarised in Table 6. These results suggest that the prognostic value of IPI was not likely driven by overestimation due to competing mortality. Nevertheless, because competing events were imputed rather than observed, the simulation findings should be interpreted with caution.

**Table 6:** Pre‑specified competing‑risks sensitivity simulation: subdistribution hazard ratios for postoperative recurrence

Parameter	sHR (95% CI)	*P* value
IPI ≥ 0.223 (vs. < 0.223)	5.91 (2.87–12.19)	**< 0.001**
Diabetes mellitus (yes vs. no)	2.41 (1.11–5.25)	**0.027**
Age (per year)	1.02 (0.99–1.05)	0.268
T stage (T1 vs. Ta)	1.70 (0.83–3.51)	0.150
Tumour number (multiple vs. single)	1.39 (0.75–2.57)	0.294
Tumour size (per cm)	1.02 (0.80–1.30)	0.867

This is a hypothetical simulation because competing‑event status was imputed from population life tables, not directly observed. The covariate set mirrors the primary Cox model (Table 4)

*sHR* Subdistribution hazard ratio, *CI* Confidence interval, *IPI* Inflammatory prognostic index

## Discussion

### Principal findings and clinical significance

To our knowledge, IPI has not previously been evaluated as a prognostic tool in patients undergoing TURBT for NMIBC. The present study therefore fills an important gap. In the pre‑specified primary analysis with IPI modelled as a continuous variable, each 0.1‑unit increase was associated with a 12% higher recurrence risk (HR 1.12, 95% CI 1.07–1.18). Consistent with this, a high preoperative IPI (dichotomised at the ROC‑derived threshold for clinical interpretability) was associated with more aggressive tumour features and independently increased the hazard of postoperative recurrence by more than fivefold. Among the four inflammatory indices examined, IPI had the best discriminatory accuracy (AUC = 0.790) and was the only marker that remained significant after multivariable adjustment. This suggests that its composite structure may capture risk information that is missed by single-ratio markers. In descriptive comparisons, CRP was substantially higher in the recurrence group (median 1.08 vs. 0.11 mg/dL, *P* < 0.001), whereas albumin was similar between groups (median 3.95 vs. 3.94 g/dL, *P* = 0.416). In this cohort, the prognostic value of IPI may therefore have been driven mainly by systemic inflammatory activity. These findings extend the possible usefulness of IPI from advanced urothelial malignancy to earlier, organ-confined disease.

### Biological rationale for composite inflammatory indices

The advantage of IPI over single-ratio markers is biologically plausible. CRP reflects hepatic acute-phase activation in response to inflammatory signals, the neutrophil-to-lymphocyte component reflects the balance between pro-tumorigenic innate inflammation and anti-tumorigenic adaptive immunity, and albumin serves as a marker of the nutritional and catabolic state associated with chronic inflammation [21–23]. When these processes occur together, they may weaken tumour immune surveillance and reduce host resilience, creating conditions that favour tumour re-establishment after surgical resection. This may explain why composite inflammatory scores, including IPI, often outperform individual ratios across tumour types [12–15]. The present results are also consistent with studies showing prognostic value for U-IPI in advanced urothelial cancer [16] and for other multi-component indices in bladder cancer, including the C-reactive protein-to-albumin ratio, the prognostic nutritional index, and the modified Glasgow Prognostic Score [24–26].

### Non-linear dose–response pattern

The non-linear relationship found in the RCS analysis is clinically relevant. Recurrence risk was relatively stable at low IPI values but rose sharply once IPI exceeded about 0.2, a value close to the ROC-derived cut-off of 0.223. This pattern suggests a threshold effect. Low-grade systemic inflammation may be compensated for by host immune surveillance, whereas inflammation above a critical level may create an immunosuppressive and pro-angiogenic environment that facilitates tumour re-implantation and outgrowth after TURBT. Clinically, this non-linearity supports the binary stratification used in this study, because the additional prognostic information from IPI appeared mainly in the elevated range. It also suggests that interventions aimed at reducing inflammation or improving general condition, such as nutritional optimization, better control of comorbidities, or selected anti-inflammatory approaches, may be most relevant for patients whose IPI is above this threshold.

### Model calibration and robustness

Model performance was also supported by calibration analysis. The Brier score was 0.091, and the bias-corrected calibration slope was 0.86. These calibration metrics are not often reported in NMIBC biomarker studies, but they are important because they indicate whether predicted risks are close to observed outcomes. The present results therefore add confidence to the potential clinical use of an IPI-based model.

#### Comparison with other inflammatory markers

Both NLR and LMR lost statistical significance after multivariable adjustment, which is consistent with the vulnerability of single-ratio markers to confounding by clinicopathological factors [11]. Some BCG-treated cohorts have found NLR to be prognostic, but differences in risk composition and treatment background may explain the discrepancy with our findings. PLR showed no significant predictive value, in keeping with the mixed evidence reported in previous studies [9].

### Complementary role of IPI to pathological stratification

The pre-specified sensitivity analyses showed that IPI remained independently significant when either pathological grade or EAU risk group was added to the model. VIF analysis suggested minimal collinearity between IPI and these pathological variables (VIF = 1.091, 2.299, and 2.251, respectively; all < 5), so the lack of significance for grade and risk group in the combined models was unlikely to be a collinearity artefact. These results indicate that IPI may provide prognostic information beyond standard histopathological assessment. Even so, IPI should be considered an adjunct to established pathological risk stratification rather than a replacement for it.

### Competing risks and study robustness

In elderly cohorts, standard Kaplan–Meier analysis can overestimate cumulative recurrence when non-cancer death competes with the event of interest. To explore whether such competing mortality could materially bias our conventional estimates, we performed a pre-specified competing-risks sensitivity simulation using the Fine–Gray subdistribution hazard model. Because all-cause mortality data were not systematically collected in this retrospective cohort, competing-event status was not directly observed but was imputed from age-specific Chinese life-table (GBD 2021) mortality rates; this analysis is therefore explicitly hypothetical rather than a formal competing-risks analysis based on empirically observed events. Under these assumptions, the subdistribution hazard ratio for IPI ≥ 0.223 (sHR 5.91, 95% CI 2.87–12.19; *P* < 0.001) was close to the primary Cox HR of 5.574, suggesting that the association between IPI and recurrence was unlikely to have been materially driven by competing non-cancer mortality. Nevertheless, because the competing events were simulated rather than observed, these results should be regarded as exploratory and interpreted with appropriate caution, and they do not substitute for a competing-risks analysis using observed mortality data.

### Diabetes mellitus as an independent predictor

Diabetes mellitus was also an independent predictor of recurrence (HR 2.853), which is consistent with meta-analytic evidence linking diabetes to adverse bladder cancer biology [27, 28]. A recent multicentre study of BCG-treated patients did not find the same association [29]. Differences in patient characteristics, treatment intensity, and follow-up may partly explain this inconsistency. Further studies are needed to clarify how metabolic comorbidities influence NMIBC recurrence.

### Clinical implications

From a practical standpoint, IPI can be calculated easily from routine preoperative laboratory tests and does not require additional testing. Patients with IPI ≥ 0.223 had substantially shorter mean RFS (18.0 vs. 33.4 months) and may benefit from closer cystoscopic surveillance or more intensive adjuvant strategies. However, the proposed threshold should be validated prospectively and externally before being used to guide clinical decisions.

### Limitations and future directions

This study has several limitations. First, the sample size was modest (*n* = 123, 44 events), which may affect the precision of the hazard ratio estimates and the stability of the optimal IPI cut‑off. Although bootstrap internal validation suggested limited overfitting (optimism = 0.020) and the events‑per‑variable ratio was acceptable, the 95% bootstrap confidence interval for the cut‑off was wide (0.084–0.617), underscoring the need for external validation in larger cohorts. With a larger sample, the recurrence rate and effect estimates might shift. Second, this was a single-centre retrospective study, and the IPI cut-off of 0.223 has not yet been externally validated. Bootstrap internal validation suggested limited overfitting (optimism = 0.020) and reasonable threshold stability (bootstrap median 0.227, 95% CI 0.084–0.617), but the wide confidence interval for the cut-off shows that independent confirmation is still needed before clinical use. Third, the small number of Tis cases (*n* = 7) precluded meaningful subgroup analysis for this population; IPI performance in pure Tis cohorts warrants separate investigation. Fourth, intravesical instillation regimens differed according to EAU risk group. Adjustment for instillation type did not materially change the prognostic value of IPI, and treatment modality was not independently associated with recurrence; nevertheless, residual confounding related to treatment intensity cannot be ruled out. Fifth, the sample size was too small for meaningful subgroup analyses by treatment regimen. Sixth, the Fine–Gray analysis was a sensitivity simulation based on imputed competing‑event status rather than observed mortality data; its results should be viewed as exploratory and not as a formal competing risks analysis. Larger prospective multicentre studies are needed to validate the proposed threshold and determine whether IPI-guided surveillance improves NMIBC management.

## Conclusions

Preoperative IPI is a simple, low-cost composite inflammatory index that was independently associated with recurrence after TURBT in this single-centre NMIBC cohort. It showed better discriminatory accuracy than NLR, PLR, and LMR and was linked to more aggressive tumour characteristics. The primary continuous analysis, the non‑linear dose–response pattern, and good model calibration support its potential role as an adjunct to existing pathological risk stratification for individualised postoperative surveillance. However, the cut-off value reported here (≥ 0.223) is cohort-specific and should not be used for clinical decision-making until it has been validated in external datasets.

## Supplementary Information

Supplementary Material 1.

## Data Availability

The datasets used and/or analysed during the current study are not publicly available due to institutional privacy policies and ethical restrictions approved by the Ethics Committee of Xingtai People’s Hospital (Approval No. 2020【147】). The data contain potentially identifiable patient information and cannot be deposited in a public repository under the terms of the ethics approval. De-identified data supporting the conclusions of this article are available from the corresponding author (ShuBo Chen; csb@xtrmyy.cn) upon reasonable request, subject to institutional approval.
