# Supplementary material for: Prognostic value of the inflammatory prognostic index for postoperative recurrence in non-muscle-invasive bladder cancer
Source: BMC Urol. 2026 Aug 7;26:275. doi: 10.1186/s12894-026-02301-3 (PMC13733249; doi:10.1186/s12894-026-02301-3)
Supplement: Supplementary file 1 [file 12894_2026_2301_MOESM1_ESM.docx]

**Supplementary Table S1** Sensitivity analysis excluding one extreme IPI outlier (IPI = 97.8)

| **Analysis** | **Including outlier (n = 123)** | **Excluding outlier (n = 122)** |
| --- | --- | --- |
| IPI, continuous (HR per 0.1‑unit increase, 95% CI) | 1.12 (1.07–1.18) | 1.13 (1.07–1.19) |
| Optimal IPI cut‑off (Youden) | 0.223 | 0.223 |
| AUC (95% CI) for continuous IPI | 0.790 (0.705–0.874) | 0.800 (0.707–0.893) |
| HR for IPI ≥ 0.223 (multivariable) | 5.574 (2.583–12.029) | 5.601 (2.595–12.089) |

Using the original cut‑off of 0.223 for consistency. All HRs are from the primary multivariable Cox model.
